# Impact of seasonal influenza on polyclinic attendances for upper respiratory tract infections in Singapore

**DOI:** 10.5365/wpsar.2019.10.4.001

**Published:** 2020-06-30

**Authors:** Annabel C.Y. Soh, Anurag Sharma, David J. Muscatello

**Affiliations:** a University of New South Wales, Kensington, New South Wales, Australia.

## Abstract

**Purpose:**

The burden of influenza on primary health-care services is not well established in tropical countries, where there are no clearly defined influenza seasons. We aimed to estimate the association between influenza infection activity and polyclinic attendance rates for upper respiratory tract infections (URTIs) in the Singapore population.

**Methods:**

We used generalized additive time series models to estimate the association between the proportion of respiratory tests positive for influenza infection in Singapore reported to the World Health Organization every week, and the population rate of polyclinic attendances in Singapore for physician-diagnosed URTI, which includes influenza-like illness (ILI), for six years from 2012 through 2017. Where data were available, we controlled for other infections that can cause fever or respiratory symptoms.

**Results:**

Influenza, dengue fever and chickenpox (varicella) were positively associated with acute URTI polyclinic attendances. The estimated URTI polyclinic attendance rates attributable to influenza, dengue fever and chickenpox were 618.9 (95% confidence interval [CI]: 501.6–736.3), 153.3 (95% CI: 16.5–290.2) and 1751.5 (95% CI: 1246.3–2256.8) per 100 000 population per year, respectively.

**Conclusion:**

Influenza poses a considerable burden on primary health-care services in Singapore. However, a substantial number of polyclinic attendances due to febrile infections such as dengue fever and chickenpox appear to be recorded as URTI in the polyclinic database. These associations require further investigation.

Recent global estimates of influenza-associated mortality are in the range of 290 000–650 000 deaths every year. (*1*) Availability of hospital admission and vital statistics databases on mean hospitalizations or deaths attributable to influenza are most often studied. (*2*) However, influenza infections leading to health care for relatively mild symptoms often go unobserved at the population level. The milder outcomes of influenza have not been fully studied despite their greater prevalence. (*3*)

Singapore is a highly developed country with strong health information systems. This, combined with its equatorial location, makes Singapore an ideal candidate for estimating influenza burden in the tropics. Singapore’s health information systems include a database of attendances at polyclinics. Polyclinics are the first point of contact that patients have with the health-care system when they present with a medical condition. There are around 20 government polyclinics that provide 20% of Singapore’s primary health care. (*4*) Patients can present to these polyclinics for the treatment of acute conditions or for the follow-up of chronic conditions. (*5*)

A widely used method to estimate the burden of influenza is the Serfling regression model. (*6*) The model was originally used to estimate influenza-attributable excess mortality from a time series of deaths classified due to pneumonia or influenza. However, one of the limitations is that the model assumes a cyclical baseline activity of influenza due to the distinct seasonality of background (non-influenza) deaths in temperate countries. (*7*, *8*) Yet this may be less applicable in tropical countries such as Singapore, where seasonality is less clearly defined. (*9*, *10*)

The generalized additive model (GAM) can be used for time series analysis that more flexibly addresses the issue of less distinct seasonality from which excess outcomes attributable to influenza can be discerned. The GAM approach models the baseline activity using a more flexible approach than the Serfling model. (*11*) Unlike Serfling’s traditional approach, which excluded influenza periods to ensure the model was not influenced by the effect of epidemics on the time series, the GAM approach requires independent variables that are a complete time series. One of these time series, a parametric component of the model, needs to reflect the changing incidence of influenza in the population over time. The GAM approach also includes a non-parametric smoothing function of time that reflects the background incidence of unmeasured causes of disease that contribute to the time series, typically a spline curve. (*7*)

The objective of this study was to estimate the burden of milder influenza infections on polyclinic attendance rates in Singapore. We used time series analysis to estimate the association between influenza and polyclinic attendances for upper respiratory tract infections (URTIs), which include influenza-like illness (ILI), from 2012 through 2017. Where data were available, we controlled for other infections that can cause fever or upper respiratory symptoms.

## Materials and Methods

### Study setting and study period

We performed a retrospective observational time series analysis of influenza infections and polyclinic attendances in Singapore for 2012 through 2017, for 313 weeks over the six-year period. The first week of 2012 was recorded as Week 1.

### Data sources

Available data relevant to URTI or ILI and other fever-causing infections were downloaded from the Singapore Government’s data portal. (*12*) These were average daily polyclinic attendances in each week with a physician diagnosis of URTI. The definition of URTI in the database includes ILI. Patients are diagnosed with URTI at the polyclinic when they present with acute upper respiratory symptoms including cough or sore throat, with or without fever (> 38 °C). Patients are diagnosed with ILI when they present only with cough and fever (> 38 °C) (Ministry of Health Singapore, personal communication, 10 July 2018). We also obtained average daily polyclinic attendances for chickenpox (varicella) as chickenpox is a possible cause of fever or upper respiratory symptoms in the early stages of infection.

Influenza surveillance data for Singapore were retrieved from the World Health Organization’s FluNet database. (*13*) The weekly number of respiratory specimens reported to FluNet that were positive for influenza was obtained. (*14*)

Weekly counts of all available infectious diseases from the Weekly Infectious Diseases Bulletin published by the Ministry of Health, Singapore, (*15*) that could produce fever or upper respiratory symptoms were obtained. The illnesses available were dengue fever, *Haemophilus influenzae* type b, legionellosis and malaria.

Polyclinics are open only for half a day on Saturdays and closed on Sundays and public holidays. Public holidays affect the hours that polyclinics are open each week and thus the number of weekly attendances. Thus, holidays were included in the model to account for their effects. The number of public holidays in each week was tabulated from press releases from the Ministry of Manpower, Singapore. (*16*) School holidays may also affect patient demand and were compiled based on publicly available information provided by the Ministry of Education, Singapore. (*17*)

### Analysis

We used a GAM to investigate the association of influenza and other infectious diseases with acute URTI polyclinic attendances. Since the daily average counts of weekly polyclinic attendances for acute URTIs were large (~3000 in 2017), a model with normally distributed residuals was assumed. GAMs can include linear parametric terms and a non-parametric, nonlinear smoothing functions of the independent variables. (*18*) A natural cubic spline of week number was used as the non-parametric smoother to account for unobserved background variation in acute URTI polyclinic attendances not associated with the included parametric, independent variables, as described previously. (*7*)

The model equation for the daily average number of acute URTI polyclinic attendances included the following variables: a model intercept; parameter estimates for all six diseases each multiplied by their respective independent variable; public holidays; school holidays; week number; and an error term. Consistent with similar studies, (*7*, *18*, *19*) the smoothing spline of week number included 36 degrees of freedom (six per year), to control for medium- and longer-term variation and seasonality of background polyclinic attendances. This effectively controlled for variation in the time series on time scales longer than two months, leaving shorter time scale variation to be explained by the independent variables in the model.

To estimate the weekly values of average daily polyclinic attendances attributable to each independent variable, the parameter estimates were multiplied by the observed value of the variable in that week. The parameter estimate of chickenpox was multiplied by the weekly number of chickenpox polyclinic attendances. The parameter estimate of influenza was multiplied by the number of positive specimens in a week. For dengue fever, its parameter estimate was multiplied by the number of infections in one week.

To obtain annual total polyclinic attendances attributable to each variable, the estimates of average daily acute URTI polyclinic attendances were multiplied by the number of days that polyclinics were open each year. Since polyclinics only operate for half a day on Saturdays and are also closed on public holidays, the number of days that polyclinics are open can vary each week. The total number of attendances each year was converted to population rates using annual population estimates. (*20*)

SAS Version 7.1 was used for data analysis. Quantile-quantile (QQ) plots were used to check the modelling assumption that the error term was normally distributed. Autocorrelation plots were used to identify autocorrelation in the error term time series, which is another modelling assumption (independence of residuals).

### Ethical approval

This project was approved by the UNSW Sydney Human Research Ethics Committee as a negligible risk project (HC number: 180169).

## Results

### Data characteristics

**Figure 1:**
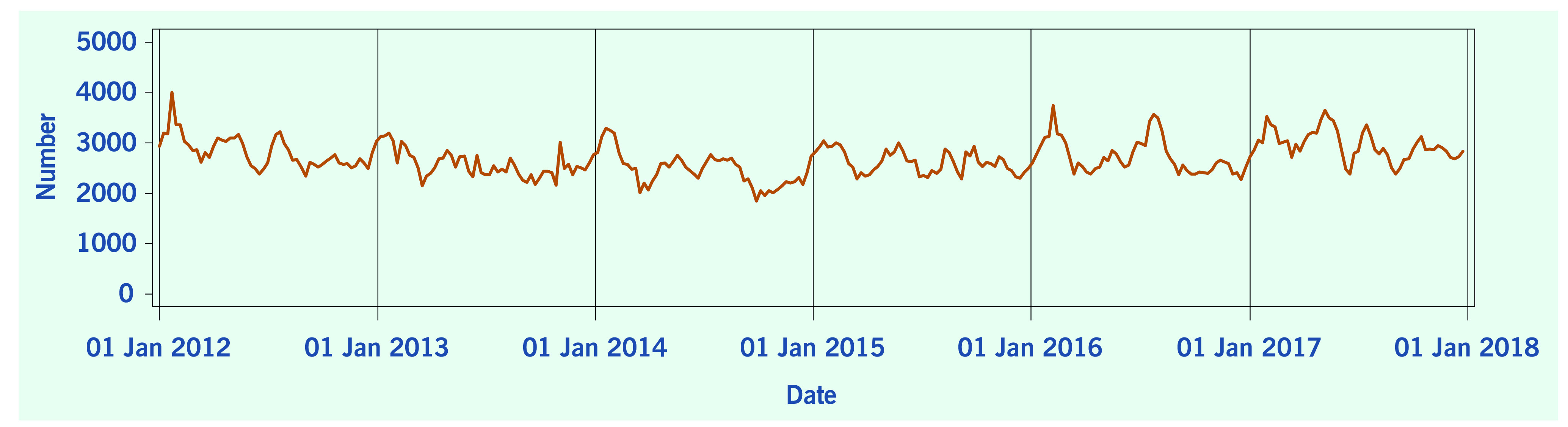
**Average daily acute URTI polyclinic attendances, by week, Singapore, 2012–2017**

**Figure 2:**
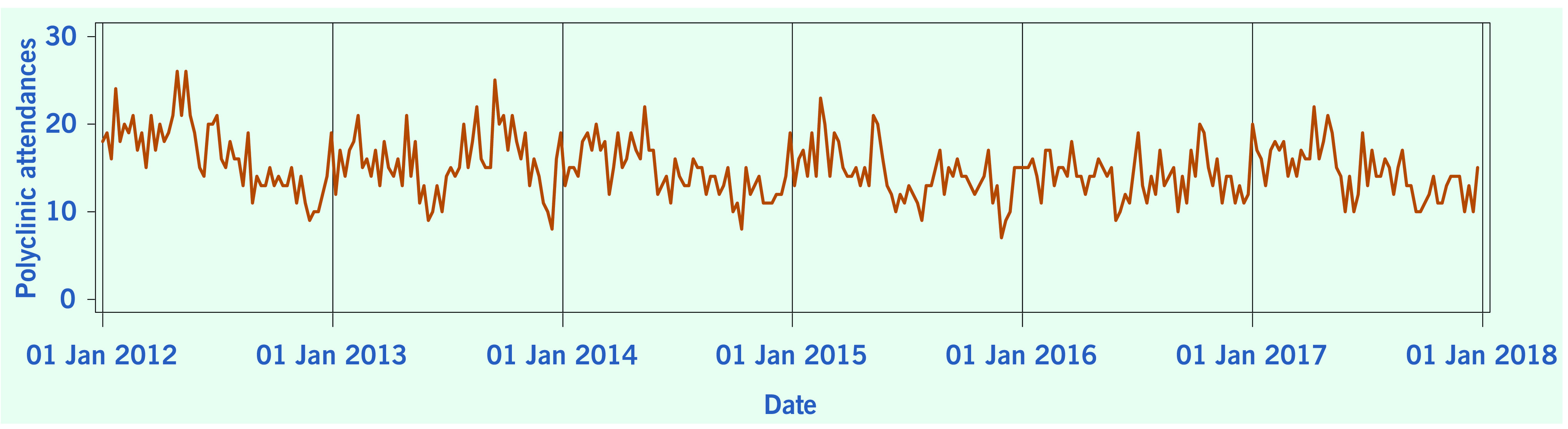
**Average daily chickenpox polyclinic attendances, by week, Singapore, 2012–2017**

**Figure 3:**
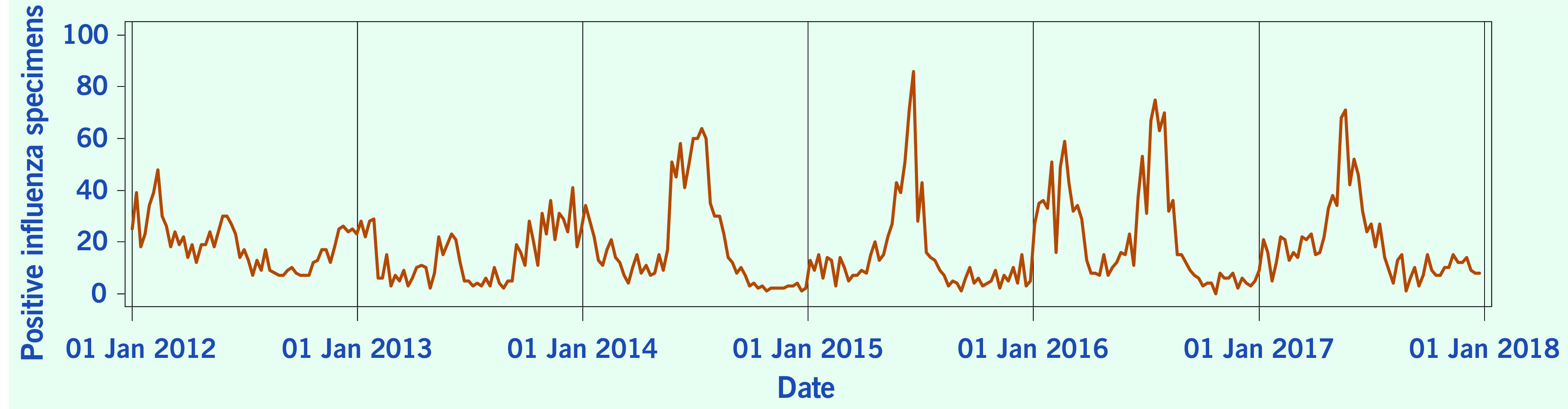
**Number of positive influenza specimens, by week, Singapore, 2012–2017**

**Figure 4:**
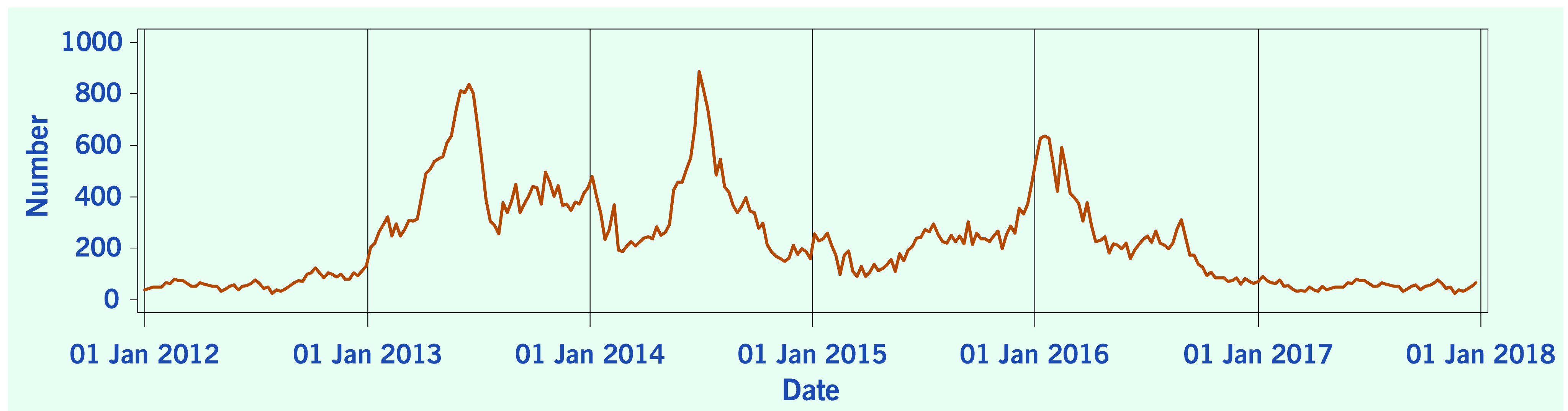
**Number of reported dengue fever infections, by week, Singapore, 2012–2017**

A total of 313 weeks from January 2012 to December 2017 was included in the study. During that period, the mean number of average daily acute URTI polyclinic attendances each week was 2694.0 (**Table 1**, **Fig. 1**). The mean number of average daily chickenpox polyclinic attendances was 15.0, and attendances occurred throughout the year without apparent seasonality (**Table 1**, **Fig. 2**). The mean number of weekly positive influenza specimens was 18.2, and infections increased during epidemic periods that varied in amplitude between years and occurred at different times of the year (**Table 1**, **Fig. 3**). The mean number of dengue infections each week was 230.7. There were dengue epidemics in some, but not all, years and background rates varied between years (**Table 1**, **Fig. 4**).

[**insert** Figure 1]

[**insert** Figure 2]

[**insert** Figure 3]

[**insert** Figure 4]

**Table 1:** Descriptive statistics of variables considered

Variable	Measured as	Mean	Median	Minimum	Maximum	Interquartile Range
URTI	Average daily polyclinic attendances each week	2694	2648	1839	4001	448
Chickenpox	Average daily polyclinic attendances each week	15	14	7	26	4
Positive influenza specimens	Weekly count	18	14	0	86	17
Dengue fever	Weekly count	231	204	24	888	233
*Haemophilus influenzae* type b	Weekly count	0	0	0	3	0
Malaria	Weekly count	1	1	0	9	2
Legionellosis	Weekly count	1	0	0	5	1

The mean number of reported *Haemophilus influenzae* type b, malaria and legionellosis cases each week were markedly lower at 0.1, 1.0 and 0.5, respectively, over the six-year period.

### Model fit

The QQ plots showed that the model assumption of a normally distributed error term was reasonable, although there were some departures from normality at the extremes. The modelling assumption of a non-autocorrelated error term was incompletely met, with some low but statistically significant autocorrelation evident in the model residuals.

We attempted to reduce this residual autocorrelation by introducing first-order autoregressive terms into the model. This may be plausible due to delayed health-care seeking following infection. The resulting model therefore included a lag term of one week for each of the three diseases. Autocorrelation in the error term was not affected by this change to the model so the autoregressive terms were discarded.

As a sensitivity analysis, we changed the functional form of the GAM to a Poisson model, with a log link function and Poisson error term. This did not alter the error term autocorrelations. Therefore, this model was discarded in favour of the simpler linear GAM model form with a normally distributed error term.

### Main results

In the initial model with all available independent variables, weekly occurrences of laboratory-confirmed influenza infections and dengue fever, and of physician-diagnosed chickenpox, were statistically significantly associated with weekly rates of acute URTI polyclinic attendances. The parameter estimates for each of these variables were 6.9 (95% CI: 5.6–8.2), 0.1 (95% CI: 0.02–0.3), and 24.0 (95% CI: 17.2–30.8), respectively (**Table 2**).

**Table 2:** Parameter estimates for each variable in the initial model

Parameter	Parameter estimate	95% confidence interval	*p*-value
Intercept	2086.7	1944.0, 2 229.4	< 0.0001
Influenza	6.9	5.6, 8.2	< 0.0001
Dengue fever	0.1	0.02, 0.3	0.03
Chickenpox	24.0	17.2, 30.8	< 0.0001
*Haemophilus * *influenzae* type b	34.0	−11.4, 79.5	0.1
Malaria	−1.4	−15.6, 12.8	0.8
Public holidays	92.1	47.8, 136.4	< 0.0001
School holidays	−208.2	−256.0, −160.4	< 0.0001
Week number	0.7	0.5, 1.0	< 0.0001

*Haemophilus influenzae* type b, legionellosis and malaria did not show a significant association with acute URTI polyclinic attendances. Due to their lack of association and extremely low frequencies, they were excluded from the final model.

In the revised model, chickenpox, influenza and dengue fever remained statistically significantly associated with the number of acute URTI polyclinic attendances. The parameter estimate for chickenpox was 23.3 (95% CI: 16.5–30.0), while the parameter estimate for influenza was 6.8 (95% CI: 5.5–8.0). The parameter estimate for dengue fever was 0.1 (95% CI: 0.01–0.2) (**Table 3**).

**Table 3:** Parameter estimates for each variable in the revised model

Parameter	Parameter estimate	95% confidence interval	*p*-value
Intercept	2105.2	1975.7, 2 234.7	< 0.0001
Influenza	6.8	5.5, 8.0	< 0.0001
Dengue fever	0.1	0.01, 0.2	0.02
Chickenpox	23.3	16.5, 30.0	< 0.0001
Public holidays	92.3	48.2, 136.4	< 0.0001
School holidays	−206.2	−253.6, −158.9	< 0.0001
Week number	0.8	0.5, 1.0	< 0.0001

**Figure 5:**
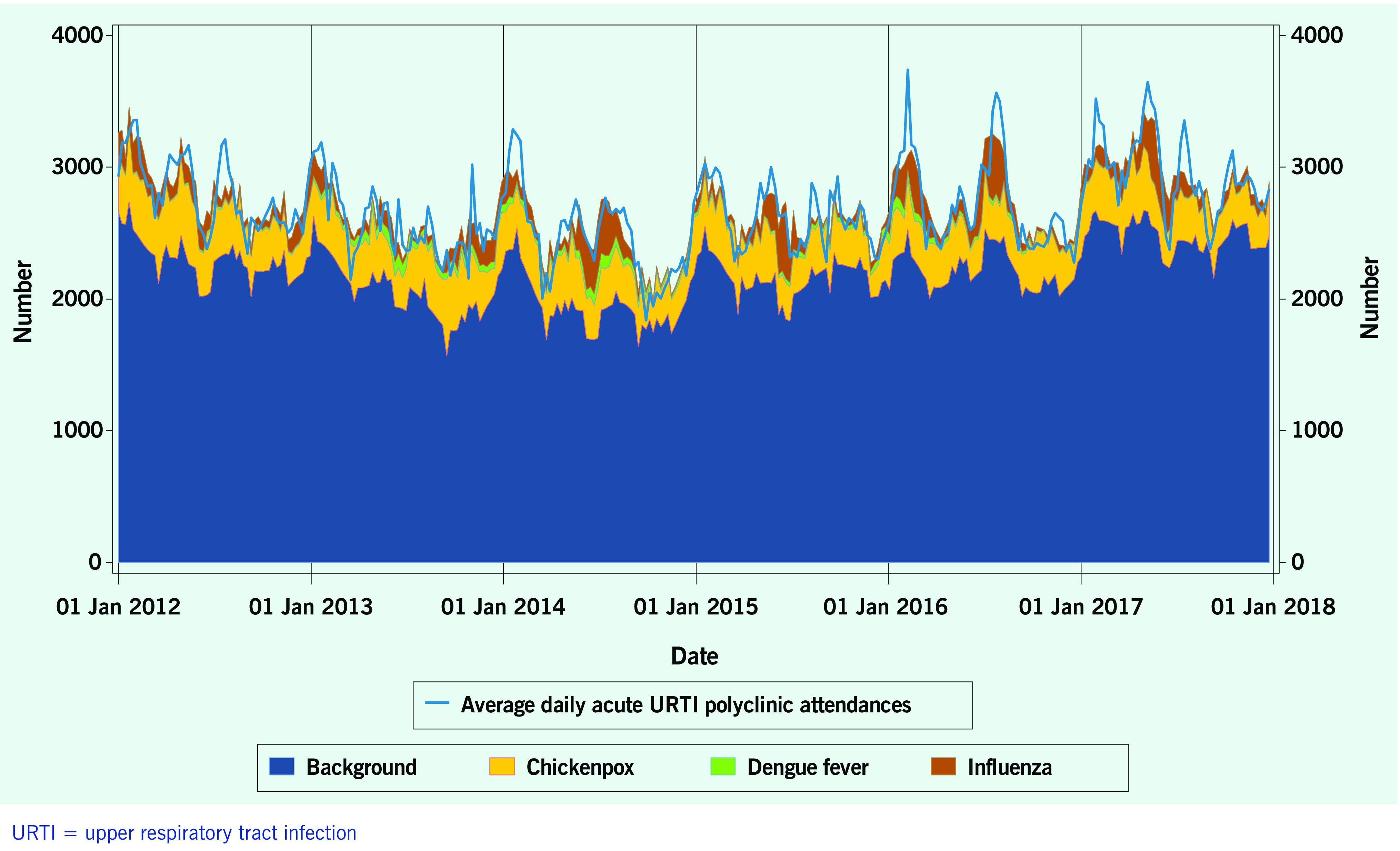
**Observed totals and estimated averages of daily URTI polyclinic attendances attributable to each disease and to background causes, by week, Singapore, 2012–2017**

The average annual estimated polyclinic attendance rate per 100 000 population was estimated to be the highest for chickenpox at 1751.5 (95% CI: 1246.3–2256.8), as compared to influenza and dengue fever at 618.9 (95% CI: 501.6–736.3) and 153.3 (95% CI: 16.5–290.2) respectively (**Table 4**, **Fig. 5**). When aggregated by year, chickenpox was estimated to constitute the greatest proportion of acute URTI polyclinic attendances across all six years. The percentage of acute URTI polyclinic attendances attributable to chickenpox, influenza and dengue fever was 13.0%, 4.6% and 1.2%, respectively, over the study period.

[**insert** Figure 5]

**Table 4:** Estimated polyclinic attendance rate per 100 000 population by disease and year, Singapore,  2012–2017

Disease	Year	Estimated attendances	95% CI	% of total URTI attendances	Rate/100 000	95% CI
Influenza	2012	35 181	28 510, 41 853	4.5	662.2	536.7, 787.8
	2013	27 071	21 938, 32 205	3.8	501.4	406.3, 596.5
	2014	35 214	28 536, 41 892	5.2	643.8	521.7, 765.9
	2015	27 751	22 488, 33 013	3.8	501.4	406.3, 596.4
	2016	43 049	34 885, 51 213	5.7	767.7	622.1, 913.3
	2017	35 755	28 974, 42 535	4.4	637.1	516.3, 757.9
	Average	34 004	27 555, 40 452	4.6	618.9	501.6, 736.3
Dengue fever	2012	3216	347, 6 085	0.4	60.5	6.5, 114.5
	2013	15 415	1661, 29 170	2.2	285.5	30.8, 540.3
	2014	12 573	1355, 23 791	1.8	229.9	24.8, 435.0
	2015	7875	849, 14 902	1.1	142.3	15.3, 269.2
	2016	9391	1012, 17 770	1.3	167.5	18.0, 316.9
	2017	1928	208, 3 648	0.2	34.4	3.7, 65.0
	Average	8400	905, 15 894	1.2	153.3	16.5, 290.2
Chickenpox	2012	106 324	75 655, 136 993	13.7	2001.4	1424.1, 2 578.7
	2013	99 859	71 055, 128 663	14.1	1849.5	1316.0, 2 383.0
	2014	93 851	66 780, 120 922	13.8	1715.8	1220.9, 2 210.7
	2015	90 759	64 580, 116 938	12.6	1639.7	1166.7, 2 112.7
	2016	91 645	65 210, 118 080	12.2	1634.4	1163.0, 2 105.8
	2017	93 634	66 626, 120 643	11.5	1668.4	1187.1, 2 149.6
	Average	96 012	68 318, 123 706	13.0	1751.5	1246.3, 2 256.8

URTI = upper respiratory tract infection

CI = confidence interval

## Discussion

This study quantifies the influenza activity associated with polyclinic attendances for acute URTIs. An estimated average of 618.9 URTI polyclinic attendances per 100 000 population per year were attributable to influenza. Assuming these polyclinic attendances represent 20% of total primary health-care episodes in Singapore with the remainder of primary care services delivered privately, (*21*) the national rate of total influenza-attributable primary care attendances may be around 3100 per 100 000. This is higher than the estimated rate of 2156 per 100 000 in England. (*22*) The percentage of URTI polyclinic attendances in Singapore that were estimated to be attributable to influenza was 4.6%, and this is also higher than the estimated 2.2% for primary care in Beijing, China, but lower than the estimated 8.7% in the United States of America. (*23*, *24*)

The total influenza burden also comprises hospitalizations and deaths in addition to primary care encounters. A study on influenza-associated deaths in Singapore found that the average estimated rate was 14.8 per 100 000 person-years from 1996 to 2003. (*25*) The rate of influenza-associated hospitalizations diagnosed with influenza or pneumonia was 29.6 per 100 000 person-years from 2010 to 2012. (*10*) This is likely to underestimate total hospitalizations attributable to influenza, which are often estimated based on broader diagnosis categories such as all respiratory diagnoses. (*26*) The rate of influenza-attributable URTI polyclinic attendances is far higher than these more severe outcomes. This is in line with the understanding that mild influenza infections constitute a large proportion of the influenza burden. (*3*, *27*)

The number of influenza-attributable polyclinic attendances dipped slightly in 2015 before rising in 2016. This may be due to the introduction of a novel influenza A(H3N2) strain to Singapore in 2016, against which the population did not have prior immunity. (*28*) Furthermore, although vaccines including one active against A(H3N2) strain were available in Singapore, there are low levels of vaccination uptake in the Singaporean population. (*29*) In addition, the reduced effectiveness of the vaccine protective against A(H3N2) vaccine virus strains could have also contributed to the increase in influenza-attributable polyclinic attendances in 2016. (*30*, *31*)

A surprising result was that both dengue fever and chickenpox were associated with acute URTI polyclinic attendances. This could be because the clinical symptoms of dengue fever and chickenpox both include fever and these diseases could therefore be mistaken for acute URTI in the early days following infection. (*32*, *33*) This highlights the discriminatory limitations of syndromic data for estimating the burden of influenza infection in primary care. (*34*, *35*) In China, Taiwan, China, it was found that predictors such as the absence of rashes, platelet count, rhinorrhoea, malaise and sore throat were useful in distinguishing influenza from dengue fever or other febrile illnesses. (*36*)

The estimated average annual rate of influenza-attributable URTI polyclinic attendances was approximately one third that of chickenpox-attributable URTI polyclinic attendances. This suggests that chickenpox may have a very high incidence compared with influenza in Singapore. Varicella is highly infectious, and a high incidence of infection has been reported in Singapore and Hong Kong Special Administrative Region SAR (China). (*30*, *37*) Varicella is not currently included in the Singapore childhood immunization schedule, but it is recommended for adults. (*38*, *39*) It is no longer a notifiable disease, but a seroprevalence study from 2008 to 2010 showed that seroprevalence of infection was around 30% in Singapore infants and around 80% by age 17 years and varicella vaccination uptake was estimated at 52%. (*40*) Thus, a high rate of infection is not surprising. The time series of chickenpox polyclinic attendances shows that chickenpox circulates throughout the year, which could also explain the relatively high estimates. Influenza and dengue fever, on the other hand, showed varying incidence over time associated with epidemic activity. The relationship of chickenpox with URTI polyclinic attendances, however, does require further investigation; it may be able to be further elaborated through age-specific analysis. (*41*)

Our results also showed that public holidays had a positive association with URTI polyclinic attendances, whereas school holidays a negative association. This is consistent with the trend shown by other infectious diseases in Singapore, where hand, foot and mouth disease demonstrated a seasonal trough during school holidays. (*42*) A European study investigating the spread of infectious diseases showed that the highest incidence of cases in an epidemic occurred in schoolchildren. (*43*) This is because contact made within this age group was more likely to be physical, and also because children tended to have a larger social circle than other age groups, leading to a greater dissemination of diseases. These reasons could therefore explain the lower rates of influenza-attributable polyclinic attendances for URTIs during school holidays in Singapore. On the other hand, mass gatherings during public holidays are likely to contribute to greater spread of diseases like influenza. (*44*)

An advantage of our study is that we were able to use a time series of laboratory-confirmed influenza infections to provide a proxy for week-to-week changes in the incidence of influenza infections in Singapore. By using only laboratory-confirmed infections, this potentially allows for a more accurate estimation of influenza-attributable URTI polyclinic attendance incidence.

The study had some limitations. Our results did not account for the mild-to-moderate influenza infections where medical care is not sought. In addition, the main limitation is that government-run polyclinics represent only around one fifth of primary care services in Singapore, with the remainder delivered privately. Thus, our results do not represent the total primary care burden of influenza in Singapore. Nevertheless, polyclinics remain an important component of the primary health-care sector in Singapore, and the database of information on polyclinic attendances can allow for a relatively continuous estimation of influenza’s burden on primary health care in the country. Also, we were unable to obtain age-specific information for this study. The data available also did not include information on lower respiratory tract infections or other common respiratory pathogens such as rhinovirus and respiratory syncytial virus. Lack of information on other sources of variation in the time series may have explained some of the low residual first-order autocorrelation (r = 0.29), despite the use of the smoothing spline. The degree of smoothing we chose was predetermined to avoid over-fitting the model. In Singapore, school holidays can vary by institution, and we used the holiday dates associated with the main educational institutions. Sick leave entitlements may influence health-care-seeking behaviour. Singaporeans are entitled to a maximum of 14 days of paid outpatient sick leave every year. (*16*) This limited entitlement may lead patients to avoid seeking treatment of relatively mild infections. Alternately, it may increase presentations because of the need to obtain a medical certificate. There is limited information on influenza vaccination levels in Singapore, although a recent estimate is 14% coverage in 65–74 year olds. (*45*) The vaccine is recommended for all residents, but it is only subsidized for employed citizens and permanent residents with a high risk for severe infection outcomes through the national medical savings scheme (MediSave). (*46*) Depending on citizenship or residency status, a polyclinic visit can cost up to 68 Singapore dollars in 2019. (*47*) This cost, with the additional cost of a vaccine, may lead to a lower vaccine uptake.

In summary, influenza may pose a considerable health-care burden on primary health-care services in Singapore. The data from our study may be helpful in supporting cost–effectiveness studies to evaluate if an influenza immunization policy would be beneficial to the Singaporean population. This could, in turn, lower the rates of polyclinic attendances for influenza. The surprising finding that a substantial proportion of URTI presentations appears to be associated with chickenpox and dengue fever activity requires further study.
